# Rapidly Fatal *Acanthamoeba* Encephalitis and Treatment of Cryoglobulinemia

**DOI:** 10.3201/eid1303.061001

**Published:** 2007-03

**Authors:** Wouter Meersseman, Katrien Lagrou, Raf Sciot, Johan de Jonckheere, Christine Haberler, Julia Walochnik, Willy E. Peetermans, Eric van Wijngaerden

**Affiliations:** * Gasthuisberg University Hospital, Leuven, Belgium; † Scientific Institute of Public Health, Brussels, Belgium; ‡ Medical University of Vienna, Vienna, Austria

**Keywords:** Acanthamoeba, encephalitis, immunosuppression, rituximab, dispatch

## Abstract

We describe a 66-year-old woman with therapy-refractory cryoglobulinemia treated with rituximab, plasmapheresis, and steroids; a case of fatal meningoencephalitis caused by *Acanthamoeba* spp. then developed. Such infections are rare and show an unusually rapid course (possibly related to rituximab).

Infection with *Acanthamoeba*, a free-living ameba, is a rare cause of slowly progressive granulomatous amebic encephalitis (GAE) in immunocompromised patients. This form of encephalitis is almost universally progressive and fatal, typically within 2 months of symptom onset (*1*). We describe a patient with cryoglobulinemia refractory to standard therapy who died of GAE after receiving rituximab.

Rituximab is a monoclonal antibody directed against CD20, a surface antigen expressed in cells of B-lymphocyte lineage. It was developed for treatment of B-cell lymphomas. Recently, therapy with rituximab has been extended to a variety of autoimmune diseases in which B cells have been thought to play a role. The drug substantially depletes normal B cells from the peripheral blood and its use leads to a prolonged period of humoral immune dysfunction (*2*).

## The Case

**Figure 1:**
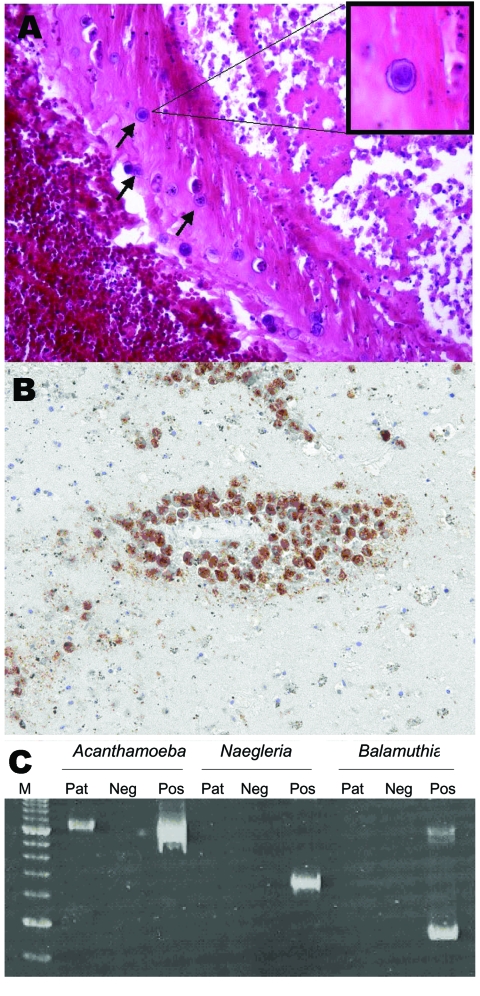
Magnetic resonance image of the patient’s brain after the first seizure showing a hemorrhagic lesion in the right frontal lobe.

In March 2005, a 66-year-old woman was referred to our hospital because of status epilepticus. A diagnosis of hepatitis C had been made in 2003. In January 2005, hepatitis C–related cryoglobulinemic vasculitis with cutaneous and renal involvement developed. The cryoprecipitate contained 586 mg/L of immunoglobulin G (IgG) and 517 mg/L of IgM (normal <10 mg/L). Treatment with prednisolone (1 g intravenously for 3 days, followed by 64 mg orally once a day until day 31) was started in January 2005 (day 1). Despite this treatment, the vasculitis worsened. Plasmapheresis was started on day 10 (8 sessions) and resumed on day 39 (10 sessions). The patient’s cryocrit decreased. The prednisolone dose was tapered on day 31 to 16 mg. Increasing palpable purpura on the lower legs was observed on day 46. The decision was made to start rituximab infusions (375 mg/m^2^), which she received starting on day 48 on a weekly basis; she received 2 infusions. On day 55, she had a seizure and was transferred to the intensive care unit. She was deeply comatose with a Glasgow coma score of 4/15. Vital signs were normal. Magnetic resonance imaging showed a T2 hyperintense lesion in the left frontal region of the brain (Figure 1). At that time, the lesion was believed to be related to the cryoglobulinemia. Examination of cerebrospinal fluid (CSF) showed a protein level of 228 mg/dL, a glucose level of 53 mg/dL, and 13 lymphocytes/µL. Culture of blood and CSF, serologic tests, and PCR did not show any fungi, viruses, or bacteria. The patient remained in a nonconvulsive status epilepticus despite combination anticonvulsive therapy. She died on day 61.

**Figure 2:**
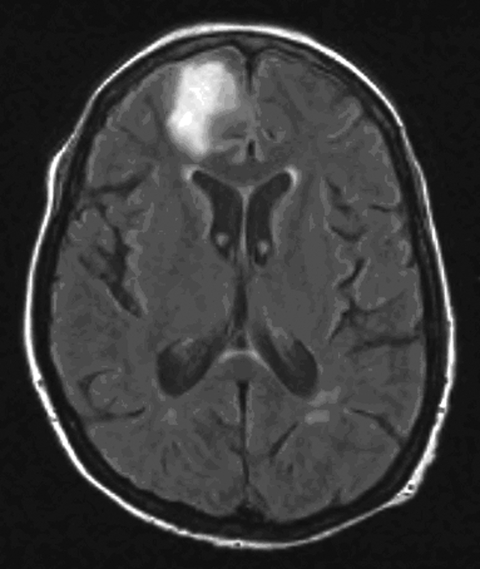
A) Cysts in a vessel wall (arrows) of the patient (hematoxylin and eosin stain, magnification ×250). Inset shows a cyst at higher magnification (hematoxylin and eosin stain, magnification ×800). B) Immunohistochemical staining with antibody to *Acanthamoeba* cysts within vessel walls (magnification ×250). C) Polyacrylamide gel electrophoresis of PCR products for *Acanthamoeba* spp., *Naegleria* spp., and *Balamuthia mandrillaris* using JDP primers for a diagnostic small subunit rDNA fragment. M, molecular mass marker; Pat, patient; Neg, negative control; Pos, positive control.

Postmortem examination showed signs of glomerulonephritis, liver fibrosis, and moderate signs of myocarditis. A formalin-fixed, 1,378-g brain specimen showed no discoloration or meningeal opacification throughout most parts of the brain. Small lacerations were visible in the pedunculus cerebri superior and both hemispheres of the cerebellum. No obvious intraparenchymal bleeding was present. Hematoxylin and eosin–stained sections showed a necrotizing hemorrhagic meningoencephalitis and adjacent recent infarctions. Numerous trophozoites and *Acanthamoeba* cysts were observed within necrotic areas and overlying meninges. Trophozoites were predominantly perivascularly located in nonnecrotic areas and cysts were detectable within blood vessel walls (Figure 2A). For immunohistochemical analysis, 3-µm–thick sections of paraffin-embedded brain tissue were cut and stained with an antibody to *Acanthamoeba* spp. (from rabbits immunized with *Acanthamoeba* genotype T4) at a dilution of 1:2,000. Antigen retrieval was performed by heating sections in 0.01 mol/L citrate buffer (pH 6.0) for 60 minutes. ChemMate kit K5001 (Dako, Glostrup, Denmark) was used for immunostaining (Figure 2B).

We conducted a PCR for detection of *Acanthamoeba* spp., *Balamuthia mandrillaris*, and *Naegleria fowleri* in brain tissue and CSF by using JDP primers for a diagnostic small subunit rDNA fragment as previously described (*3*). *Acanthamoeba* DNA was detected in brain tissue but not in CSF. Typing of the *Acanthamoeba* strain by sequencing the amplified partial small subunit rRNA gene region (ASA.S1) with primer 892c as previously described (*4*) showed that the strain had genotype T4. The sequenced region is identical to that found in the European Molecular Biology Laboratory database (accession no. AP07407 (Figure 2C).

## Conclusions

We report a rapidly fatal case of GAE in a woman with cryoglobulinemia treated with rituximab. GAE is a rare but often lethal subacute cause of meningoencephalitis, typically occurring in immunocompromised patients. *Acanthamoeba* spp. are the most common free-living amebae, are ubiquitous in the environment, and are widespread in water and soil. Low-level antibody titers are found in >50% of asymptomatic patients (*5*,*6*). More than 20 species of these amebae have been described (*1*). In addition to causing keratitis after use of contact lenses, *Acanthamoeba* spp. are responsible for meningoencephalitis in chronically ill, debilitated patients, in patients taking immunosuppressive drugs or receiving chemotherapy, and in patients with AIDS (*1*). GAE shows a chronic course with atypical symptoms of low-grade fever, encephalopathy with cognitive abnormalities, headache, and seizures. This disease is rarely identified in patients before death. CSF analysis shows moderate pleiocytosis, but it rarely contributes to the diagnosis because amebae are generally not found in the CSF. Recently, detection of serum antibodies was successfully used in screening for free-living amebae in patients in California with encephalitis (*7*). However, this process proved to be of value mainly for identifying *B*. *mandrillaris*, another free-living ameba.

Evidence of *Acanthamoeba* infection was recently found by PCR in brain tissue of a patient with lupus, even without isolating the ameba (*8*). A PCR with tissue and CSF is potentially useful in clinical laboratories in identifying the cause of meningoencephalitis without the need for specific antibodies.

Because the patient’s cryoglobulinemia was poorly controlled, we decided to begin treating our patient’s condition with rituximab. This monoclonal antibody has been shown to be effective in treating cryoglobulinemia resistant to standard therapies with corticosteroids and plasmapheresis (*9*). This antibody against the CD20 receptor on the surface of the B lymphocytes destroys these cells by mechanisms involving complement-mediated and antibody-dependent cytotoxicity (*10*). Levels of B cells remain low for 2 to 6 months, which leads to long-term humoral immune dysfunction. Serious viral infections such as erythrocyte aplasia caused by parvovirus B19, fatal hepatitis B, and cytomegalovirus and varicella zoster virus infections have been reported after administration of rituximab (*11*–*14*). The fact that our patient died of *Acanthamoeba* encephalitis is striking because rituximab is believed to interfere with humoral immunity, which is not known to play a major role against free-living amebic infections.

We identified a temporal relationship between the weekly rituximab treatments and meningoencephalitis. Although the patient received corticosteroids and underwent plasmapheresis, GAE developed only after rituximab infusions had begun and the steroid dose was being tapered. It is not clear whether rituximab is the only agent responsible for the GAE, or what contributions were made by the earlier treatments the patient received. We could not identify the source of infection in this patient. In particular, we found no evidence for nosocomial acquisition. No other patient had a diagnosis of *Acanthamoeba* brain infection, and no one receiving rituximab had unexplained meningoencephalitis in 2005 in our institution. Our hypothesis is that *Acanthamoeba* spp. were already present in the brain at the time of the first rituximab infusion, but that rituximab may have precipitated the unusually rapid course of the encephalitis.

Rituximab is obtaining widespread use in hematologic and autoimmune diseases as an adjuvant therapy. The full spectrum of opportunistic infections in patients receiving combinations of immunosuppressive regimens remains to be elucidated and warrants vigilance.

## References

[1] Schuster FL, Visvesvara GS. Free-living amoebae as opportunistic and non-opportunistic pathogens of humans and animals. Int J Parasitol. 2004;34:1001–27. 10.1016/j.ijpara.2004.06.004 15313128

[2] Eisenberg R. Update on rituximab. Ann Rheum Dis. 2005;64(Suppl 4):iv55–7. 10.1136/ard.2005.042648 16239389 PMC1766904

[3] Foreman O, Sykes J, Ball L, Yang N, de Cock H. Disseminated infection with *Balamuthia mandrillaris* in a dog. Vet Pathol. 2004;41:506–10. 10.1354/vp.41-5-506 15347823

[4] Booton GC, Visvesvara GS, Byers TJ, Kelly DJ, Fuerst PA. Identification and distribution of *Acanthamoeba* species genotypes associated with nonkeratitis infections. J Clin Microbiol. 2005;43:1689–93. 10.1128/JCM.43.4.1689-1693.2005 15814986 PMC1081337

[5] Cerva L. *Acanthamoeba culbertsoni* and *Naegleria fowleri*: occurrence of antibodies in man. J Hyg Epidemiol Microbiol Immunol. 1989;33:99–103. 2723426

[6] Chappell CL, Wright JA, Coletta M, Newsome AL. Standardized methods of measuring *Acanthamoeba* antibodies in sera from healthy human subjects. Clin Diagn Lab Immunol. 2001;8:724–30. 11427418 10.1128/CDLI.8.4.724-730.2001 PMC96134

[7] Schuster FL, Honarmand S, Visvesvara GS, Glaser CA. Detection of antibodies against free-ling amoebae *Balamuthia mandrillaris* and *Acanthamoeba* species in a population of patients with encephalitis. Clin Infect Dis. 2006;42:1260–5. 10.1086/503037 16586385

[8] Shirwadkar CG, Samant R, Sankhe M, Deshpande R, Yagi S, Schuster FL, *Acanthamoeba* encephalitis in patient with systemic lupus, India. Emerg Infect Dis. 2006;12:984–6. 16707057 10.3201/eid1206.060087 PMC3373031

[9] Zaja F, de Vita S, Mazzaro C, Sacco S, Damiani D, de Marchi G, Efficacy and safety of rituximab in type II mixed cryoglobulinemia. Blood. 2003;101:3827–34. 10.1182/blood-2002-09-2856 12560225

[10] Johnson P, The mechanisms of action of rituximab in the elimination of tumor cells. Semin Oncol. 2003;30(Suppl 2):3–8. 10.1053/sonc.2003.50025 12652458

[11] Sharma VR, Fleming DR, Slone SP. Pure red cell aplasia due to parvovirus B19 in a patient treated with rituximab. Blood. 2000;96:1184–6. 10910942

[12] Dervite I, Hober D, Morel P. Acute hepatitis B in a patient with antibodies to hepatitis B surface antigen who was receiving rituximab. N Engl J Med. 2001;344:68–9. 10.1056/NEJM200101043440120 11187122

[13] Suzan F, Ammor M, Ribrag V. Fatal reactivation of cytomegalovirus infection after use of rituximab for a post-transplantation lymphoproliferative disorder. N Engl J Med. 2001;345:1000. 10.1056/NEJM200109273451315 11575282

[14] Bermudez A, Marco F, Conde E, Mazo E, Recio M, Zubizarreta A. Fatal visceral varicella-zoster infection following rituximab and chemotherapy treatment in a patient with follicular lymphoma. Haematologica. 2000;85:894–5. 10942955

